# Efficacy of Jackson-Pratt Mediastinal Drains in Reducing Pericardial Effusion and Atrial Fibrillation After Coronary Artery Bypass Grafting: A Retrospective Cohort Study

**DOI:** 10.21470/1678-9741-2025-0277

**Published:** 2026-02-18

**Authors:** Mehmet Ali Yuruk, Ahmet Coşkun Özdemir

**Affiliations:** 1 Cardiovascular Surgery, Faculty of Medicine, Karadeniz Technical University, Trabzon, Turkey

**Keywords:** Chest Tubes, Coronary Artery Bypass Grafting, Jackson-Pratt Drains, Cardiac Tamponade, Atrial Fibrillation.

## Abstract

**Introduction:**

Postoperative complications such as pericardial and pleural effusions, cardiac tamponade, and atrial fibrillation (AF) are common after coronary artery bypass grafting (CABG). While standard chest tubes are routinely used for drainage, Jackson-Pratt drains (JP-D) may offer advantages due to their flexible design and ability to maintain negative pressure.

**Methods:**

This retrospective study compared outcomes between patients who received conventional chest tubes drains (CT-D group) (n = 672; 2016 - 2020) and those who received JP-D in addition to standard drains (JP-D group, n = 706; 2020 - 2023) after CABG. Demographic, operative, and postoperative data were collected and analyzed.

**Results:**

Both groups were similar in baseline characteristics (P > 0.05 for all). The JP-D group had significantly lower rates of cardiac tamponade (0.28% vs. 1.78%, P = 0.008), reoperation (1.55% vs. 4.61%, P = 0.001), wound infections (2.1% vs. 4.1%, P = 0.024), 30-day mortality (1.1% vs. 2.0%, P = 0.035), and postoperative AF (9.2% vs. 16.8%, P = 0.039). Despite a higher first-day drainage volume (480 ± 150 mL vs. 360 ± 120 mL, P = 0.030), total drainage volume was similar. Pulmonary complications, including atelectasis and pneumonia, were also significantly reduced in the JP-D group.

**Conclusions:**

The use of JP-D in conjunction with standard thoracic drainage after CABG was associated with improved postoperative outcomes, including reduced effusion-related complications and AF. These findings suggest potential benefits of JP-D in cardiac surgery, though prospective studies are warranted to confirm these results.

## INTRODUCTION

**Table:** 

Abbreviations, Acronyms & Symbols	
AF	= Atrial fibrillation
BSA	= Body surface area
CABG	= Coronary artery bypass grafting
COPD	= Chronic obstructive pulmonary disease
CT	= Computed tomography
CT-D	= Chest tube drains
EF	= Ejection fraction
EuroSCORE	= European System for Cardiac Operative Risk Evaluation
JP-D	= Jackson-Pratt drains
SD	= Standard deviation

Chest tubes are routinely placed following cardiac surgery to facilitate fluid drainage from the thoracic cavity, enable early detection of postoperative bleeding, prevent cardiac tamponade, and reduce the risk of postoperative pleural effusion or pneumothorax - particularly when the pleural space is entered during open-heart procedures or removing the left internal thoracic artery graft. Depending on the evaluation methods and diagnostic criteria used, pericardial effusion following cardiac surgery has an incidence ranging from 1% to 85%, while delayed cardiac tamponade may occur in up to 15% of cases^[1,2]^. Atrial fibrillation (AF) is frequently associated with pericardial effusion and cardiac tamponade and has been linked to prolonged hospital stays and higher readmission rates^[3]^. To address these complications, thick, long, and semi-rigid chest drains are traditionally employed. However, these drains are often associated with frequent administration of sedatives and analgesics, which may contribute to hypoventilation and subsequent atelectasis^[4]^.

Several studies have demonstrated that small, flexible silicone drains are comparable in efficacy to larger drains following cardiac surgery^[5]^. Jackson-Pratt drains (JP-D) (Ethicon, United States of America), characterized by soft silastic tubing with four lateral channels surrounding a solid central core, offer a lower risk of obstruction due to their flexibility and reduced diameter. Evidence suggests that JP-D may be more effective than conventional chest tube drains (CT-D) in reducing the incidence of postoperative complications such as AF, pericardial effusion, and cardiac tamponade^[6]^. However, the literature presents some variability, with certain studies indicating that JP-D may be less effective than larger drains^[7]^.

In this study, we hypothesized that JP-D, as an alternative to standard-sized chest drains following open-heart surgery, could reduce the incidence of pericardial effusion, pleural effusion, cardiac tamponade, and AF while maintaining the efficacy of postoperative fluid drainage.

## METHODS

CT-D were used in 672 patients (CT-D group) who underwent coronary bypass surgery via median sternotomy between January 2016 and December 2020, while JP-D were used in 706 patients (JP-D group) operated on between January 2020 and December 2023. Demographic and clinical data, including age, sex, body surface area, and other relevant characteristics, were analyzed for all patients in both groups. The study was approved by the Ethics Committee of Karadeniz Technical University, adhering to the principles outlined in the Declaration of Helsinki (Ethics Committee Approval No: 2022/212).

Patients aged between 18 and 75 years who underwent open-heart surgery and primary coronary artery bypass grafting (CABG) were included in the study. Exclusion criteria were the presence of congenital heart disease, prior reoperation, additional surgical procedures (such as valve surgery), patients with a history of AF who are receiving medical treatment, and the administration of preoperative anticoagulant therapy. Data for all patients were collected through a retrospective review of medical records.

Preoperative preparation, anesthesia management, cardiopulmonary bypass procedures, and postoperative care protocols were standardized and consistent across both groups. In the CT-D group, a standard thoracic drain was placed in the mediastinal and pleural cavities. In the JP-D group, in addition to the standard thoracic drains, a JP-D was inserted into the mediastinum, positioned posterior to the heart. Drains were kept in place until the drainage volume was reduced to 50 mL.

Preoperative, operative, and postoperative data were collected from patient records, including demographic characteristics, clinical features, surgical details, and postoperative outcomes. During the wound care and follow-up period, complications were assessed through physical examination. Infections were identified based on culture results and clinical signs. Pericardial fluid accumulation was distinguished from pleural effusion when assessing infection, as it serves as an early indicator for potential complications that could arise later.

Complications:

a. Cardiac tamponade: Postoperatively, all patients were monitored daily with chest radiography. Cardiac tamponade was diagnosed with bedside echocardiography in patients with mediastinal widening and clinical symptoms (hypotension, tachycardia, tachypnea, decreased urine output). In cases of unclear, confirmation was made with thoracic computer tomography. All patients with tamponade underwent intervention.
b. AF: All patients who had a new-onset AF attack during postoperative hospital follow-up, documented with an electrocardiogram, and started medical treatment were considered to have AF.
c. Atelectasis: It was detected by daily chest radiography and physical examination, and in patients in whom a definitive diagnosis could not be made, a differential diagnosis was made by computerized thorax tomography.
d. Pneumonia: Patients who were clinically suspected of having pneumonia (high fever, leukocytosis, high C-reactive protein, infiltration on chest radiography) were consulted to the chest diseases department, and patients who started antibiotic therapy were included in the study as having pneumonia.

### Statistical Analysis

Statistical analysis was conducted using IBM SPSS Statistics for Windows, version 25.0 (IBM Corp., Armonk, N.Y., USA). The demographic and clinical characteristics of the groups were compared using the Chi-square test. For the analysis of continuous variables, the independent sample *t*-test was employed. A significance level of *P* < 0.05 was set to evaluate the impact of the drain on time-related outcomes.

## RESULTS

The demographic characteristics of the patients involved in the study were as follows: the mean age was similar between groups (62.2 ± 8.0 *vs.* 63.6 ± 8.3 years, *P* = 0.741); the number of male patients was higher in both groups; the sex distribution was similar across both groups (*P* = 0.836); hypertension was the most common condition (CT-D: 57.2%, JP-D: 59.7%, *P* = 0.583), followed by diabetes (CT-D: 36.4%, JP-D: 38.5%, *P* = 0.624). European System for Cardiac Operative Risk Evaluation II values (CT-D: 2.8 ± 1.2, JP-D: 2.9 ± 1.3, *P* = 0.725) and preoperative ejection fraction values (CT-D: 52.5 ± 8.4%, JP-D: 51.8 ± 8.7%, *P* = 0.558) were similar in both groups, and no significant difference was found in this respect (Table 1).

**Table 1:** Demographic and clinical characteristics.

Preoperative Variables	CT-D Group	JP-D Group	*P*-value
	(n = 672)	(n = 706)	
Age, years	62.2 ± 8.0	63.6 ± 8.3	0.741
Sex, male/female, n (%)	153/519 (22.7)	166/540 (23.6)	0.836
BSA, m^2^	1.84 ± 0.22	1.86 ± 0.24	0.642
Hypertension, n (%)	385 (57.2)	422 (59.7)	0.583
Diabetes, n (%)	245 (36.4)	272 (38.5)	0.624
EuroSCORE II	2.8 ± 1.2	2.9 ± 1.3	0.725
Preoperative EF, %	52.5 ± 8.4	51.8 ± 8.7	0.558
Chronic renal failure, n (%)	84 (12.5)	97 (13.7)	0.684
COPD, n (%)	126 (18.7)	142 (20.1)	0.725
Using beta-blockers, n (%)	440 (65.4)	483 (68.7)	0.246

Values are given as mean ± standard deviation, median (interquartile range), or n (%)

BSA=body surface area; COPD=chronic obstructive pulmonary disease; CT-D=chest tube drain; EF=ejection fraction; EuroSCORE=European

System for Cardiac Operative Risk Evaluation; JP-D=Jackson-Pratt drain

No statistically significant difference was observed between the groups in terms of the intensive care unit and hospitalization durations. Patients in both groups were discharged at similar times (Table 2).

**Table 2:** Clinical outcomes belong to operation and complications.

	CT-D Group	JP-D Group	*P*-value
	(n = 672)	(n = 706)	
CABG, n (%)	672	706	0.892
Operation time (min)	190 (120 - 240)	180 (115 - 255)	0.853
Cross-clamping time (min)	45 (32 - 86)	52 (40 - 92)	0.767
Hospitalization, days (min-max)	6 (5 - 7)	7 (6 - 8)	0.701
Intensive care unit stay, hours (min-max)	40 (36 - 120)	48 (40 - 144)	0.620
Complications			
Tamponade, n (%)	12 (1.78)	2 (0.28)	0.008
Reoperation, n (%)	31 (4.61)	11 (1.55)	0.001
Transfusion requirement, n (%)	105 (15.6)	75 (10.6)	0.002
Wound site infection, n (%)	28 (4.1)	15 (2.1)	0.024
30-day mortality, n (%)	14 (2.0)	8 (1.1)	0.035
Hospital readmission, n (%)	42 (6.2)	30 (4.2)	0.042

Values are given as mean ± standard deviation or n (%)

CABG=coronary artery bypass grafting; CT-D=chest tube drain; JP-D=Jackson-Pratt drain

Regarding clinical outcomes and postoperative complications, the JP-D group had significantly lower rates of cardiac tamponade (1.78% *vs.* 0.28%, *P* = 0.008) and reoperation (4.61% *vs.* 1.55%, *P* = 0.001) than the CT-D group. The need for transfusions was also lower in the JP-D group (15.6% *vs.* 10.6%, *P* = 0.002). Additionally, the JP-D group had significantly lower rates of wound site infections (4.1% *vs.* 2.1%, *P* = 0.024), 30-day mortality (2.0% *vs.* 1.1%, *P* = 0.035), and hospital readmissions (6.2% *vs.* 4.2%, *P* = 0.042) (Table 2).

A detailed analysis of effusion and drainage data revealed that, although the JP-D group exhibited a significantly higher volume of drainage in the first 24 hours than the CT-D group (360 ± 120 ml *vs.* 480 ± 150 ml, *P* = 0.030), the total drainage volume did not differ significantly between the groups (700 ± 200 ml *vs.* 720 ± 220 ml, *P* = 0.070). Pulmonary complications were less frequent in patients within the JP-D group. Specifically, the incidences of atelectasis (6.2% *vs.* 4.2%, *P* = 0.030), pneumonia (3.1% *vs.* 2.1%, *P* = 0.040), and the requirement for pleural fluid aspiration (5.2% *vs.* 3.1%, *P* = 0.020) were significantly lower in this group. Furthermore, the JP-D group demonstrated reduced rates of pericardial effusion on postoperative echocardiography (8.3% *vs.* 5.2%, *P* = 0.010) and a lower need for thoracic computed tomography (7.2% *vs.* 4.2%, *P* = 0.020) (Table 3).

**Table 3:** Postoperative clinical data.

	CT-D Group (n = 672)	JP-D Group (n = 706)	*P*-value
First 24-hour drainage, mL (mean ± SD)	360 ± 120	480 ± 150	0.030
Total drainage, mL (mean ± SD)	700 ± 200	720 ± 220	0.070
Drain removal time, hours (mean ± SD)	60 ± 18	72 ± 24	0.020
Pulmonary complications			
Atelectasis, n (%)	42 (6.2)	30 (4.2)	0.030
Pneumonia, n (%)	21 (3.1)	15 (2.1)	0.040
Pleural fluid puncture need, n (%)	35 (5.2)	22 (3.1)	0.020
Follow-up parameters			
Effusion on postoperative echocardiography, n (%)	56 (8.3)	37 (5.2)	0.010
Need for thorax CT, n (%)	49 (7.2)	30 (4.2)	0.020
Atrial fibrillation, n (%)	113 (16.8)	65 (9.2)	0.039

Values are given as mean ± standard deviation (SD) or n (%)

CT=computed tomography; CT-D=chest tube drain; JP-D=Jackson-Pratt drain

As for the postoperative AF rates, statistically significantly less AF was observed in the JP-D group (16.8% *vs.* 9.2%, *P* = 0.039).

## DISCUSSION

This study evaluated the efficacy of JP-D on pleural and pericardial effusions, wound infections, respiratory complications, and the incidence of AF following open-heart surgery in comparison to conventional thoracic drains. The results demonstrated statistically significant differences in managing postoperative complications and overall patient recovery. Specifically, JP-D was associated with improved outcomes in several key areas. Patients in the JP-D group exhibited reduced fluid accumulation, lower rates of AF, and decreased need for reoperation, suggesting a potential clinical advantage of JP-D in the postoperative management of cardiac surgery patients.

Pleural and pericardial effusions are among the most common complications following CABG. Conventional rigid thoracic drains are often insufficient to evacuate fluid accumulation completely in the pericardial space. In contrast, the JP drains in our surgical protocol were strategically positioned posteriorly between the heart and the pericardium, allowing for more efficient drainage. Our study demonstrated that the JP drain group exhibited significantly greater drainage volumes within the first 24 hours postoperatively, which was associated with improved prevention of effusions. Yamani et al.^[8]^ (2022) emphasized the importance of effective drainage in the management of pericardial effusion, recommending that drainage be continued until the output is reduced to below 20 - 30 mL over 24 hours. Our protocol involved the removal of all drains after 24 hours, once the drainage volume had declined to < 50 mL per day.

Complications such as pericardial effusion, tamponade, and AF are among the most common complications following CABG. In our study, a significant reduction in postoperative complication rates was observed in the JP-D group, a finding that is consistent with previous reports in the literature. Stremmel et al.^[9]^ reported that continuous drainage was associated with significantly lower rates of re-tamponade (12.5% *vs.* 50.0%) and conversion to open-heart surgery (0.0% *vs.* 28.6%) and additionally demonstrated that closed drainage systems reduce the risk of infection. Similarly, Khan et al.^[10]^ found that the incidence of late cardiac tamponade was significantly lower among patients managed with an extended chest tube drainage protocol (3.6% *vs.* 8.8%). Our findings suggest that JP-D enhances the prevention of cardiac tamponade and pleural effusion by facilitating more effective fluid evacuation through continuous negative pressure around the heart.

Postoperative wound infections were less frequent in the surgical cohort managed with JP-D. This observation aligns with findings reported by Bustamante-Munguira et al.^[11]^, who identified an overall surgical site infection rate of 4.6% and noted that 13% of patients with infections required reoperation. Similarly, Zukowska et al.^[12]^ reported that the incidence of superficial wound infections following cardiac surgery ranged from 0.5% to 8%, while deep infections occurred in 0.5% to 5.6% of cases. In our study, the lower incidence of surgical site infections in the JP-D group may be attributed to the reduced occurrence of postoperative complications, including cardiac tamponade, pleural effusion, and the necessity for reoperation. The effectiveness of effusion drainage using conventional drains is influenced by multiple factors, including drain diameter, structural design, gravitational effects, and negative pressure. In the study by Lattoo et al.^[13]^, the drain-based complication rate was lower in the JP-D catheter (35.8% *vs.* 57.7%). Similarly, the occlusion rates were significantly in favor of the JP-D (7.4% *vs.* 21.9%). In a meta-analysis conducted by Wang et al.^[14]^, pressure stabilization was identified as a more critical factor than drain duration in determining drainage efficiency, with no significant differences observed in total drainage volume. In a randomized controlled trial by St-Onge et al.^[15]^, active tube cleaning was associated with a significant reduction in both cardiac tamponade (0.4% *vs.* 3.4%, *P* = 0.02) and reoperation rates (1.6% *vs.* 5.7%, *P* = 0.01). In our study, those in the JP-D group exhibited a significantly higher drainage volume within the first 24 hours postoperatively. This is likely attributable to the design features of the JP-D, including its flexible tubing, multiple drainage ports, and capacity to maintain negative pressure. However, the total amount of drainage was similar in both groups.

In terms of respiratory complications, our study found fewer occurrences in the JP-D group, like the reduced rates of effusion evacuation and wound complications. A previous study reported that 30-72% of postoperative cardiac surgery patients developed atelectasis, and 2-20% experienced pneumonia^[16]^. In our study, respiratory complications were less frequent in the JP-D group than these reported rates, likely due to more effective fluid drainage and a lower incidence of reinterventions.

An observational study by Rabelo et al.^[17]^ demonstrated that posterior pericardial chest tube drainage was associated with a significant reduction in postoperative AF. In their meta-analysis, Xiong et al.^[18]^ found an increase in the incidence of AF (10.3%) in patients who underwent posterior pericardiotomy, compared to 25.7% in the no-procedure group. Rong et al.^[19]^ found fewer AF episodes in patients who underwent posterior pericardiotomy. In our study, the incidence of postoperative AF was notably lower in the JP-D group. Additionally, AF was more prevalent in the CT-D group, likely due to the higher incidence of effusion and tamponade, which required further intervention.

### Limitations

The retrospective nature of our study, which is a major limitation, introduces potential biases such as preference bias and limits the ability to control for confounders. Another limitation of the study is that preoperative and postoperative hemoglobin levels cannot be compared. Our data indicated that patients in the JP-D group experienced fewer reoperations and better fluid management than those in the CT-D group. These findings align with previous studies, highlighting the critical role of drainage system selection in postoperative recovery and the prevention of complications. However, these insights also emphasize the need for cautious interpretation of our results. Future research employing a prospective study design with a more stringent control of variables is warranted to provide definitive evidence on the most effective drainage methods for open-heart surgery.

## CONCLUSION

This study highlights the advantages of JP-D over traditional chest tubes in managing postoperative complications after open-heart surgery. Our findings suggest that patients who underwent Jackson-Pratt drainage experienced fewer pericardial and pleural effusions, a reduction in cardiac tamponade, and fewer reoperations, indicating the effectiveness of this method in fluid management. Despite these improvements, there was no significant reduction in the length of hospital stay. Given the observational design of the current study, future research should employ prospective study designs to validate these findings and allow for more precise adjustments to surgical and postoperative protocols, ultimately enhancing patient outcomes following open-heart surgery. Nevertheless, our results offer a novel perspective on the potential benefits of incorporating JP-D into postoperative care protocols following open-heart surgery, suggesting opportunities for review and optimization of current practices.

## Data Availability

The authors declare that the data supporting the findings of this study are available within the article.

## Artificial Intelligence Usage

The authors declare that no artificial intelligence tool was used in the preparation of this article.
